# SPARC Is a New Myeloid-Derived Suppressor Cell Marker Licensing Suppressive Activities

**DOI:** 10.3389/fimmu.2019.01369

**Published:** 2019-06-20

**Authors:** Sabina Sangaletti, Giovanna Talarico, Claudia Chiodoni, Barbara Cappetti, Laura Botti, Paola Portararo, Alessandro Gulino, Francesca Maria Consonni, Antonio Sica, Giovanni Randon, Massimo Di Nicola, Claudio Tripodo, Mario P. Colombo

**Affiliations:** ^1^Molecular Immunology Unit, Department of Research, Fondazione IRCCS Istituto Nazionale dei Tumori di Milano, Milan, Italy; ^2^Tumor Immunology Unit, University of Palermo, Palermo, Italy; ^3^Department of Inflammation and Immunology, Humanitas Clinical and Research Center, Milan, Italy; ^4^Department of Pharmaceutical Sciences, University of Eastern Piedmont, A. Avogadro, Novara, Italy; ^5^Medical Oncology Department, Fondazione IRCCS Istituto Nazionale dei Tumori di Milano, Milan, Italy

**Keywords:** SPARC, myeloid-derived suppressor cells, breast cancer, neutrophil, neutrophil extracellular traps

## Abstract

Myeloid-derived suppressor cells (MDSC) are well-known key negative regulators of the immune response during tumor growth, however scattered is the knowledge of their capacity to influence and adapt to the different tumor microenvironments and of the markers that identify those capacities. Here we show that the secreted protein acidic and rich in cysteine (SPARC) identifies in both human and mouse MDSC with immune suppressive capacity and pro-tumoral activities including the induction of epithelial-to-mesenchymal transition (EMT) and angiogenesis. In mice the genetic deletion of SPARC reduced MDSC immune suppression and reverted EMT. *Spar*c^−/−^ MDSC were less suppressive overall and the granulocytic fraction was more prone to extrude neutrophil extracellular traps (NET). Surprisingly, arginase-I and NOS2, whose expression can be controlled by STAT3, were not down-regulated in *Spar*c^−/−^ MDSC, although less suppressive than wild type (WT) counterpart. Flow cytometry analysis showed equal phosphorylation of STAT3 but reduced ROS production that was associated with reduced nuclear translocation of the NF-kB p50 subunit in *Spar*c^−/−^ than WT MDSC. The limited p50 in nuclei reduce the formation of the immunosuppressive p50:p50 homodimers in favor of the p65:p50 inflammatory heterodimers. Supporting this hypothesis, the production of TNF by *Spar*c^−/−^ MDSC was significantly higher than by WT MDSC. Although associated with tumor-induced chronic inflammation, TNF, if produced at high doses, becomes a key factor in mediating tumor rejection. Therefore, it is foreseeable that an unbalance in TNF production could skew MDSC toward an inflammatory, anti-tumor phenotype. Notably, TNF is also required for inflammation-driven NETosis. The high level of TNF in *Spar*c^−/−^ MDSC might explain their increased spontaneous NET formation as that we detected both *in vitro* and *in vivo*, in association with signs of endothelial damage. We propose SPARC as a new potential marker of MDSC, in both human and mouse, with the additional feature of controlling MDSC suppressive activity while preventing an excessive inflammatory state through the control of NF-kB signaling pathway.

## Introduction

Tumor growth implies a systemic state of immune suppression, also characterized by bone marrow (BM) expansion and circulation of myeloid cells able to suppress adaptive immune responses through a variety of mechanisms (1, 2). The so-called myeloid-derived suppressor cells (MDSC) are a heterogeneous pool of myeloid cells, mainly composed by two subsets, the monocytic (M)-MDSC and the polymorphonuclear (PMN)-MDSC, characterized by different phenotypic markers, which are also distinct between human and mouse. The two subsets expand differently and are endowed with different suppressive activities depending on the specific tumor types (3, 4). Persistent tumor release of growth factors and cytokines (such as G-CSF, GM-CSF, and VEGF), promote MDSC production in the BM, whereas tumor release of chemokines (i.e., CCL2, CXCL12) recruits them within the tumor microenvironment (TME) (5, 6). Once in the TME, MDSC acquire suppressive activity through the chronic sensing of inflammatory cytokines and damage-associated molecular patterns (DAMP). PMN-MDSC are phenotypically almost indistinguishable from neutrophils, which also share several functions in favor of tumor growth and dissemination. M-MDSC are instead similar to monocytes and are characterized by high plasticity: in the TME they can differentiate in macrophages and dendritic cells (7, 8).

Neutrophils can chaperone circulating tumor cells that, through a VCAM1-mediated embrace, gain proliferative capacity while in circulation (9). Notably similar interaction, but via beta1 integrin, is retained by PMN even when dying of NETosis, a peculiar type of cell death releasing neutrophils extracellular traps (NETs) (10). NETs are double strand DNA threads decorated with anti-microbial proteins that are extruded by neutrophils to control bacterial and fungi infections. Several stimuli (e.g., IFN, TNF, IL-8, and DAMP) initiate NETosis by binding to neutrophil receptors (e.g., Fc receptors, TLRs) (11). An aberrant NET production has been reported in autoimmune conditions, such as systemic vasculitis and systemic lupus erythematosus (12). Many papers are now describing NET in the context of cancer (10, 13). In solid tumors NET have been shown in clinical samples of triple-negative human breast cancer (TNBC). Using murine models of TNBC Park et al. showed that NET stimulate invasion and migration of breast cancer cells. Inhibiting NET formation or digesting NET with DNAse I *in vivo* reduced lung metastasis. Furthermore, NET can wrap circulating tumor cells (CTC) through a β1 integrin-mediated mechanism or promote cancer cell awakening through extracellular matrix (ECM) remodeling (14, 15). It remains undetermined whether NETs, which are decorated with proteolytic enzymes active on endothelial cells in case of vasculitis, can leave tumor cells unhurt upon their contact.

In this context, we have published that inflammatory neutrophils isolated from subcutaneous agar implants spontaneously extrude NETs (16) and display cytostatic activity against cultured tumor cells (17).

Tumor growth is also associated with aberrant extracellular matrix (ECM) deposition. An increase in collagen content enhances ECM stiffness with consequences on tumor cell survival and migration (18). Other than contributing to the biological and clinical heterogeneity of solid cancers the ECM can directly affect tumor cell as well as immune cell behavior within the TME (19). When activated, immune cells express the ITIM-receptor LAIR-1 that specifically binds to Gly-Pro-Hyp collagen conserved motives (20). The triggering of this receptor activates a negative inhibitory signal that blocks cell activation including NET formation and ROS production (21, 22) We have recently demonstrated that an aberrant ECM deposition characterized by secreted protein acidic and rich in cysteine (SPARC) and high collagen content promotes the recruitment of suppressive myeloid cells (23). SPARC belongs to the class of matricellular proteins with regulatory functions tuning different biological processes, including migration, proliferation, adhesion and cell survival (24). Although not strictly endowed with structural functions, matricellular proteins can control ECM stiffness and composition. In *Spar*c^−/−^ mice, collagen fibers are smaller and disorganized (25, 26) whereas SPARC overexpression promotes collagen fiber deposition and increases ECM stiffness, which in turn activates the process of MDSC recruitment in the TME (23). These data indicate that an ECM rich in SPARC can modulate myeloid cell functions. Less studied and appreciated is the role of matricellular proteins when produced directly by myeloid cells. On this line we have previously shown that another matricellular protein, osteopontin (OPN), when produced by M-MDSC as intracellular protein (iOPN), tunes MDSC suppressive function, modulating the expression of arginase-1, IL-6 and phospho-Stat3 (27). Here we studied the role of SPARC as marker of human and mouse MDSC and its possible regulatory function on their activity.

## Materials and Methods

### Animals, Cell Lines, and *in vivo* Experiments

BALB/cAnNCrl mice (BALB/c) were purchased from Charles River Laboratories (Calco). All experiments involving animals were approved by the Ministry of Health (INT 16_2016, authorization number 288/2017-PR). *Spar*c^−/−^ mice on a BALB/c background were obtained in our laboratory as previously described (25). The mammary carcinoma cell line SN25A was obtained from SPARC-deficient mice that spontaneously developed mammary tumors due to the expression of the rat HER2/neu oncogene (BALB/c; SPARC < tm1Hwe > Tg(MMTV-Erbb2)NK1Mul/J), whereas the N3D cell line was derived from transgenic Her2/Neu mice (BALB/c-Tg(MMTV-Erbb2)NK1Mul/J). Both cell lines were infected with the retroviral vector LXSPARCSH to over-express SPARC and the co-isogenic cell lines, SN25ASP and N3DSP, were obtained (23). Mice were injected into the mammary fat pad with SN25A, N3D, N3DSP (all at the dose of 2x10^*^5 cells) and SN25ASP (10^*^6 cells) cell lines and tumors collected when they reached a 10 mm diameter.

### Patient Samples and Gene Expression Data

Peripheral Blood was obtained from consecutive breast cancer patients (12 cases) to be surgically resected at Fondazione IRCCS Istituto Nazionale Tumori. The study was approved by the Medical Ethics Committee (Auth. Number 167/17), and all clinical data were obtained after receiving informed consent, according to institutional rules. Confocal microscopy analysis was performed onto consecutive primary breast tumors surgically resected at Fondazione IRCCS Istituto Nazionale dei Tumori.

### Flow Cytometry Analysis

For FACS analysis primary tumors or spleens were collected and maintained in DMEM−10%FBS, then minced and filtered through a 40 μm-pores cell strainer (BD). Red blood cells were removed using ACK lysis buffer (ammonium chloride potassium). Cells were Fc-blocked using CD16/32 antibody (eBioscience) before staining. Antibodies used were: CD45.2; Gr-1; CD11b; Ly6G, and Ly6C (all from eBioscience). Samples were acquired using a BD LSR II Fortessa instrument and analyzed with FlowJo software (TreeStar). All samples are analyzed in single; in each experiment at least 3–4 samples were analyzed for each group.

### PBMC Flow Cytometry and Cell Sorting

Blood samples were collected in heparin and peripheral blood mononuclear cells (PBMCs) were obtained by diluting whole blood samples patients 1:2 with PBS 1X and subsequently subjected to a density gradient stratification. Briefly, diluted whole blood samples was carefully layered onto Histopaque-1077 Ficoll (Sigma- Aldrich) and centrifuged at 1,800 rpm for 30 min at room temperature without brake. Finally, the lymphocyte-enriched ring at the interface was transferred into a new collection tube and washed with PBS 1X by centrifugation at 1,200 rpm for 5 min. PBMCs were then stained and analyzed by BD LSR II Fortessa instrument. For MDSC characterization 10^6^ PBMC were stained with the following Ab: Lin1 (FITC); HLA-DR (APC eFl780), CD11b (BB700), CD14 (FITC), CD15 (BV650), CD16 (Pe-Cy7), and CD33 (PE) (Supplementary Table 1). Total MDSC were sorted from PBMC according to HLA-DR, CD33 and CD11b expression. Cells were sorted using a FACSAria BD Instrument.

### MDSC Isolation From Spleen and *in vivo* Tumors for RT-PCR

For MDSC isolation, spleens and mammary lesions from tumor-bearing mice (or naive mice as controls for spleen MDSC), were, minced and filtered to obtain a single cell suspension. Red blood cells were lysed by ACK lysis buffer and MDSC were sorted with FACSAria BD Instrument with the following antibodies: CD45, CD11b, Ly6G, and Ly6C (all from Ebioscience).

### RNA Extraction and RT-PCR

For quantitative RT-PCR, myeloid cells were lysed with TRIzol (Invitrogen Life Technologies) and RNA was extracted using the RNeasy Kit (Qiagen). DNA contaminants were removed by treatment with DNase I. cDNA was reverse transcripted from 1 μg of total RNA. PCR was performed using TaqMan Universal PCR master mix (Applied Biosystems, Foster City, CA, USA) and a target gene assay mix containing sequence-specific primers for the *Arginase1, Sparc, TGFb, TNF, NOS-2*, and *Stat3* genes. Gene-specific primers were purchased from Applied Biosystems (Tnf Mm00443260_g1; Arg Mm00475989; Stat3 Mm01219775_m1; Sparc Mm00486332_m1; Vegf Mm01281449; Nos2 Mm00440502_m1; Gapdh Mm99999915_g1). The reactions were set up according to the standard TaqMan qPCR conditions reported in the Applied Biosytems protocol and were performed in duplicate for each sample. The qPCR assays were run using the ABI PRISM® 7900 Fast Real Time PCR system and the ABI PRISM 7900 HT Sequence Detection System (Applied Biosystems), and the result data were analyzed with SDS Software 2.3 (Applied Biosystems). The mRNA level of the target genes was quantified by measuring the CT value to determine its relative expression. The results are reported using the fold change in the gene expression of the target genes relative to the internal control gene (GAPDH). The mean-fold change in target gene expression was calculated as 2-ΔΔCT, where DDCT = [(CT,Target - CT,GAPDH)sample-(CT,Target - CT,GAPDH)] internal control.

### Immunohistochemistry and Immunofluorescence

Histological and immunohistochemistry analyses of human and mouse tissues were performed as described previously (23). All antibodies that have been used are listed in Supplementary Table 2. For double-marker immunofluorescence stainings in which primary antibodies of the same made were adopted, the tyramide signal amplification system Opal multiplex IHC kit (Lot number 2395285, PerkinElmer Inc.) was adopted. Briefly, after deparaffinization, antigen retrieval was performed using microwave heating and a pH9 buffer and the first primary antibody was incubated overnight at 4°C (monoclonal anti-Human Osteonectin/Sparc, Clone ON1-1, 1:500, Life technologies). Immunofluorescence labeling was achieved by incubating with a specific secondary antibody, followed by the addition of one selected Opal fluorophore and microwave treatment in pH9 buffer. The same procedure was repeated for the second primary antibody for 90 min at room temperature (monoclonal anti-Human CD33, Clone PWS44, 1:100, Novocastra), using a different Opal fluoprophore and DAPI nuclear counterstain. All the sections were analyzed under Zeiss Axio Scope A1 optical microscope (Zeiss, Germany) and microphotographs were collected using an Axiocam 503 Color digital camera with the ZEN2 imaging software (Zeiss Germany).

### *In vitro* Suppressive Assay

Myeloid derived suppressor cells were purified using CD11b-conjugated microbeads (for overall population) and Myeloid-Derived Suppressor Cell Isolation Kit [for separation of the two subsets (Miltenyi Biotec)] following the manufacturer's instructions.

For *in vitro* suppression assay, 4 ×105 naïve BALB/c splenocytes have been labeled with CFSE (Carboxyfluorescein Succinimidyl ester; SIGMA Aldrich) and co-cultured with the different MDSC population at different ratio in presence of 2 μg/ml of soluble anti-CD3 and 1 μg/ml of anti-CD28 to activate lymphocytes. Each sample was seeded in triplicate. Proliferation of CD4 and CD8 T cells has been assessed 2 and 3 days later, by flow cytometry evaluating CFSE dilution in the CD4+ and CD8+ gated populations. Results are shown as percentage of proliferated cells.

### ROS Detection

The detection of ROS was performed on the overall population of myeloid derived suppressor cells purified using CD11b-conjugated microbeads using the CellROX® Green Reagent (Life technologies)a fluorogenic probe for measuring ROS in live cells. Oxidation of the cell-permeant dye by ROS generate a bright green fluorescence detectable at FACS.

### Evaluation of p50 and p65 Nuclear Translocation

To assess p50 and p65 nuclear translocation BM-derived MDSC were seeded onto poly-D-lysine coated glasses for 2 h in presence of TM supernatants or LPS (10 ng/ml).

Cell permeabilization was obtained after 1 h incubation with PBS 0.1% Triton-X100 (Sigma-Aldrich) plus 5% normal goat serum (Dako Cytomation, Carpinteria, CA USA) and 2% BSA, (Amersham Biosciences, Piscataway Township, NJ USA). Cells were then incubated with rabbit anti-mouse p50 NF-kB (NLS, sc-114; Santa Cruz) or rabbit anti-mouse p65 NF-kB (c-20, sc-372; Santa Cruz). After 1h of incubation at RT, goat anti rabbit AlexaFluor 488 conjugated (LifeTechnologies) were used as secondary antibodies. Nuclei were counterstained with DAPI (Invitrogen, Molecular Probes). Samples were mounted with FluorPreserve Reagent (Calbiochem San Diego, CA USA) and analyzed with a Leica SP8 I laser scanning confocal microscope using a fine focusing oil immersion lens (40X, N.A. 1.3) at 1 Airy Unit resolution and operating in channel mode with 405 and 633 nm excitations. The mean fluorescence intensity of the nucleus was quantified after a freehand drawing considering the nucleus as regions of interest using Image-pro Premium 9.2.

### *In vitro* PMN Cytostasis Assay

PMN were collected from blocks of 2% agarose and 0.2% gelatin in saline 5 days after subcutaneous implant, as described (17). PMN-mediated cytostasis was evaluated in a spectrophotometric assay in 96-well microplates to be read on a microplate spectophotometer. Briefly, after 72 h culture. cells were fixed with 5% formalin and stained with 1% methylene blue in 0.01 M borate buffer, pH 8.5. After eluting the dye from cells with 0.1 N HCl, absorbance was read at 620 nm. The percentage of growth inhibition was calculated as [1–(A-B-C)/(D-C)] ×100, where A, B, C, and D are absorbance of cultures of tumor cells and PMN, of PMN alone, of 10^*^4 target cells after adhesion for 2 h, and of the dye in wells containing tumor cells cultivated for 72 hr. Results are presented as mean (+SD) of three to six replicates.

### Statistical Analysis

Statistical analysis of single treatments was performed using the Mann-Whitney *t*-test. The significance of different combined treatments was assessed through one-way ANOVA with Dunn's multiple comparison test. For other analyses related to MDSC frequency or ELISA data, differences between groups were tested for significance using a two-tailed unpaired *t*-test. Values were considered statistically significant at *p* < 0.05. All of the analyses were performed using Prism software Version 5.0d (GraphPad).

## Results

### Frequency of MDSC in High-Grade Breast Cancer Patients and Their Expression of SPARC

**Figure 1:**
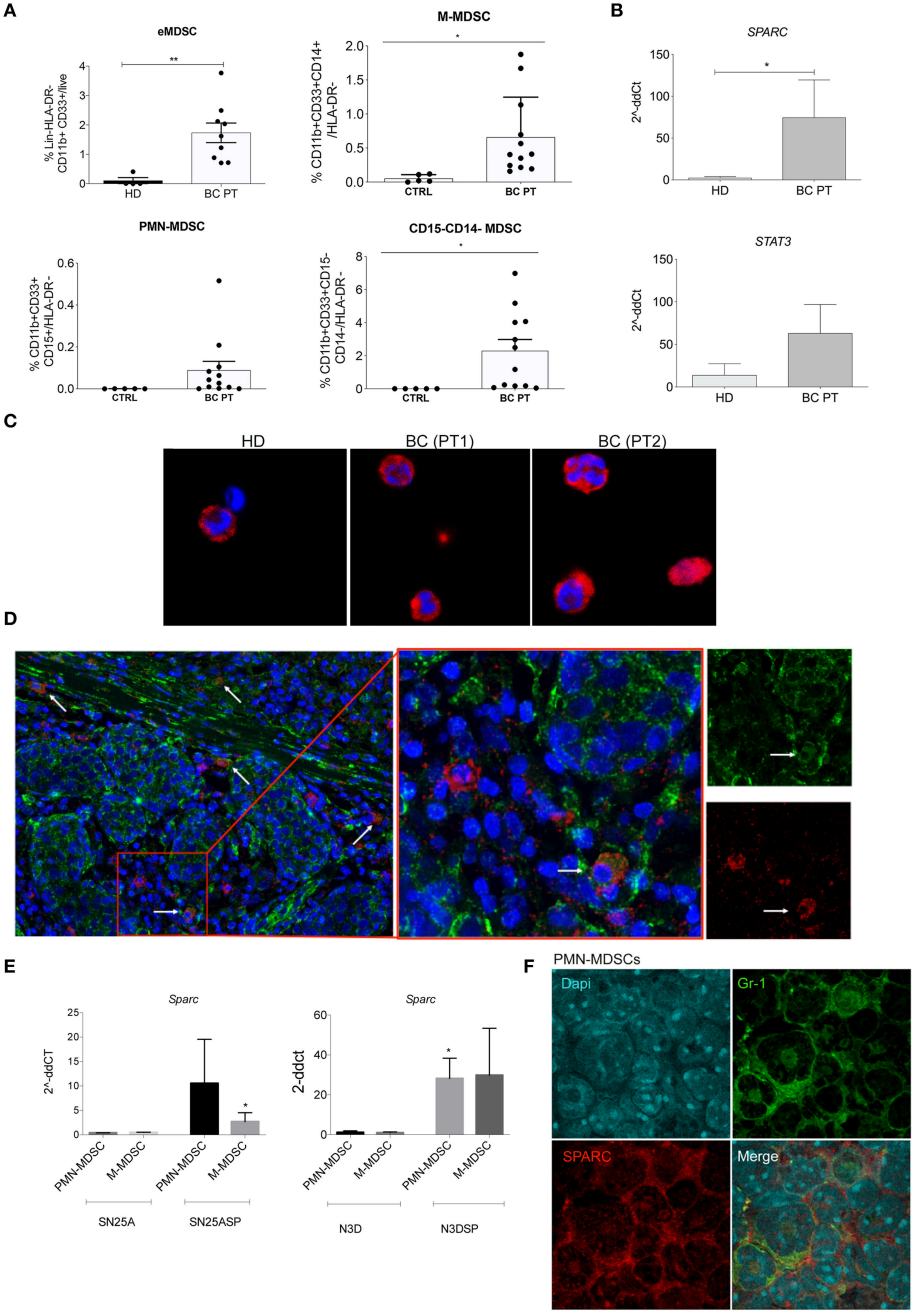
SPARC marks human and murine MDSC. **(A)** Cumulative FACS analysis showing the frequency of early MDSC (eMDSC), PMN- and M-MDSC, and CD11b+CD33+CD14-CD15- in the PB of 12 consecutive BC patients. CD11b+CD33+ eMDSC were defined within HLA-DR-Lin- cell gate. The frequency of e-MDSC was calculated as frequency of CD11b+CD33+ x Frequency of HLA-DR-/100. HLA-DR-CD33+CD11b+CD15+ PMN-MDSC and HLA-DR-CD33+CD11b+CD14+ M-MDSC were identified within the CD11b+CD33+ gate. The CD11b+CD33+ gate was defined on HLA-DR- cells. HLA-DR+ cells were identified within the gate of live cells after doublets exclusion. The frequency of PMN and M-MDSC was calculated as Frequency of CD11b+CD33+ x Frequency of CD14+ or CD15+/100. The gating strategies are shown in Supplementary Figure 1D. **(B)** Semiquantitative real-time PCR analysis for *SPARC* and *STAT3* performed on FACS-sorted MDSC isolated from breast cancer patients (BC PT; *n* = 5) compared to healthy donors (HD; *n* = 6); **(C)** Representative confocal microscopy analysis showing SPARC (red) expression in FACS-sorted HLA-DR-CD33+CD11b+ cells from two representative BC patients and one healthy control. **(D)** Representative confocal microscopy analysis for SPARC (green) and CD33 (red) showing the presence of CD33^+^ cells expressing SPARC in representative BC patient paraffin sections (white arrows). One representative case is shown. Additional cases are shown in Supplementary Figure 1. **(E)** Semiquantitative real-time PCR analysis for *Sparc* performed on murine MDSC subsets sorted from SN25A, SN25ASP, N3D and N3DSP tumors. The Student's *t*-test was used for statistical analysis (*p < 0.05; **p < 0.01). **(F)** Cytospin preparations of FACS-sorted PMN-MDSC isolated from SN25ASP tumors and stained for Gr1 (green) and SPARC (red). The same staining for M-MDSC is shown in Supplementary Figure 1C.

The peripheral blood (PB) of high-grade breast cancer (BC) patients (*n* = 12) was analyzed for the frequency of early-stage MDSC (eMDSC), identified, by flow cytometry as Lin-HLA-DR-CD33+CD11b+ [as described in (3)]. We found that high-grade BC patients have a significantly increased frequency of eMDSC if compared to healthy donors (HD) (Figure 1A and Supplementary Figure 1A for gating strategy). Using a different flow cytometry panel that includes HLA-DR, CD33, CD11b, CD14, and CD15 (3, 28) it is possible to define the PMN and M-MDSC subsets in BC patients, being PMN-MDSC HLA-DR-, CD33+CD11b+CD15+ and M-MDSC HLA-DR-CD33+CD11b+CD14+. According to this panel we found that the vast majority of MDSC expanded in BC patients are HLA-DR-CD33+CD11b+ CD14-CD15- therefore not expressing the differentiation markers (Figure 1A and Supplementary Figure 1B for gating strategy). However, the few M-MDSC were increased in BC patients compared to HD (Figure 1A).

To assess the expression of SPARC in circulating human MDSC total HLA-DR-CD33+CD11b+ MDSC were FACS-sorted and evaluated, by real-time (RT)-PCR and confocal microscopy, for the expression of SPARC at RNA and protein level, respectively (Figures 1B,C). RT-PCR analysis showed that the expression of SPARC was significantly higher in MDSC obtained from BC patients compared to HD (Figure 1B). In line, confocal microscopy analysis confirmed SPARC expression in MDSC of BC patients and less on the fewer HLA-DR-CD33+CD11b+ cells obtained from one HD (Figure 1C). To evaluate whether human myeloid cells express SPARC *in situ* in the tumor microenvironment (TME) we performed a double staining confocal microscopy analysis of BC paraffin sections. The representative picture in Figure 1D shows SPARC expression in CD33+ cells within the TME.

Next we moved to mouse models to assess whether also murine MDSC express SPARC. To perform this analysis we used 4 different mouse mammary tumors, previously characterized for their different capacity to promote MDSC expansion and activation (23). Indeed, these models were used to demonstrate that SPARC over-expression in BC cells support MDSC development and expansion. In detail, SPARC-deficient (SN25A) or low expressing (N3D) did not promoted MDSC expansion, whereas the SPARC-transduced counterparts (SN25ASP and N3DSP) strongly supported MDSC recruitment and suppressive capacity (23). Using RT-PCR (Figure 1E) and immunofluorescence (IF, Figure 1F and Supplementary Figure 1C, for the M-MDSC subset) analyses we show that SPARC is expressed by both PMN- and M-MDSC dependently on concomitant tumor expression of SPARC, being associate to N3DSP and SN25ASP, but not to N3D and SN25A tumors. These data point to SPARC as a potential new marker for MDSC. Notably, human BC samples in which SPARC was absent on tumor cells were also devoid of CD33+ cells expressing SPARC, in parallel with mouse results (Supplementary Figure 1D).

### Myeloid-Derived SPARC Is Required for Epithelial-to-Mesenchymal Transition

To determine the relevance of SPARC when directly produced by MDSC we took advantage from our SN25ASP and N3DSP models in which we showed that the recruitment of MDSC activates an EMT program *in vivo* but not *in vitro* (23).

**Figure 2:**
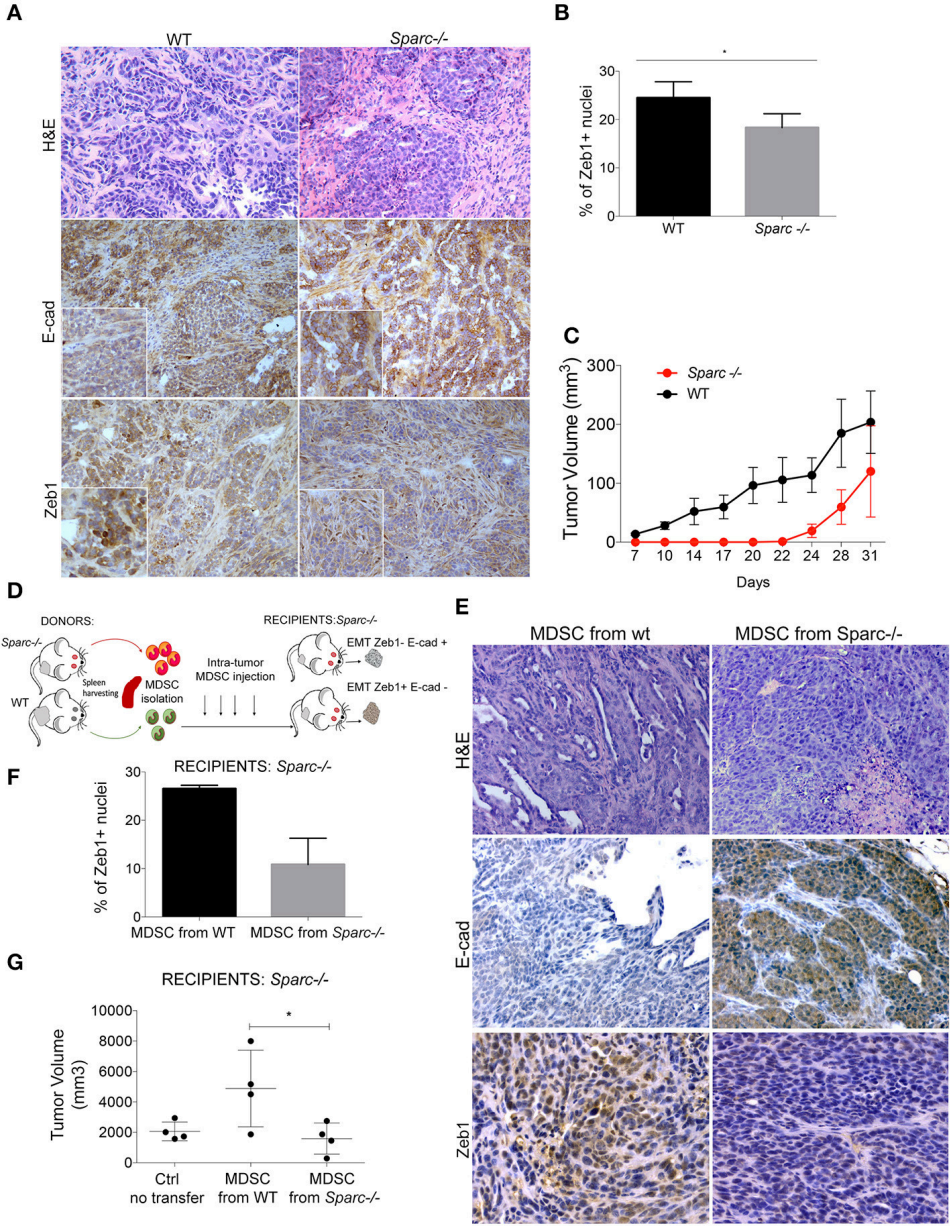
SPARC from MDSC supports EMT. **(A)** H&E and IHC analysis for E-cadherin and Zeb-1 markers performed in SN25ASP tumors obtained from WT and *Spar*c^−/−^ mice. Scale bars, 100 μm. **(B)** Quantitative IHC data for EMT markers are shown as the fraction of positive nuclei for Zeb-1 (*p < 0.05; Unpaired *T*-test) in tumors. **(C)** Mean tumor volume of SN25ASP tumors injected in WT and *Spar*c^−/−^ mice. **(D)** Graphical abstract for the MDSC transfer experiment. **(E)** H&E and IHC analysis for E-Cad and ZEB-1 showing the increased expression of EMT markers in SN25ASP tumors grown in *Spar*c^−/−^ mice transferred with WT but not *Sparc*^−/-^ MDSC. **(F)** Quantitative IHC data for EMT markers are shown as the fraction of positive nuclei for Zeb-1. **(G)** Tumor Volume of SN25ASP tumors grown in *Spar*c^−/−^ mice transferred with MDSC from WT and SPARC-deficient mice.

SPARC-producing SN25ASP cells were injected into SPARC-competent (WT) and SPARC-deficient (*Spar*c^−/−^) mice. Histopathological analysis showed that tumors developing into *Spar*c^−/−^ mice had reduced EMT features than those growing into WT recipients (Figures 2A,B). Indeed, in WT mice the tumor mass was composed mainly by cells with spindle morphology intermingled with abundant collagenic matrix forming ill-defined nest-like infiltrates. On the contrary, the EMT phenotype was almost entirely reverted in tumors grown into *Spar*c^−/−^ hosts that showed well-formed nest-like ytumor structures stained for membrane-expressed E-cadherin and reduced frequency of ZEB-1^+^ nuclei (Figures 2A,B). These data demonstrated that the robust EMT observed in SN25ASP tumors grown in WT mice was likely dependent on microenvironment-derived SPARC. Notably, SN25ASP tumors grew significantly less in *Spar*c^−/−^ than WT mice (Figure 2C), although in presence of reduced EMT. To test whether SPARC produced endogenously by MDSC contributed to EMT, 10^6^ MDSC isolated from the spleen of SN25ASP tumor-bearing WT or *Spar*c^−/−^ mice, were injected, once a week for 4 consecutive weeks (Figure 2D), intra-tumorally into SN25ASP lesions grown in *Spar*c^−/−^ mice. Results show that SN25ASP tumors gained the EMT marker ZEB-1 and lost E-cadherin, thanks to the supplement of SPARC-producing MDSC, despite the SPARC-deficiency in the host (Figures 2E,F). In presence of WT MDSC we observed also an increased tumor growth (Figure 2G). The data support the hypothesis that SPARC from MDSC is required for immune-mediated EMT.

### SPARC Specifies PMN-MDSC Suppressive Functions

**Figure 3:**
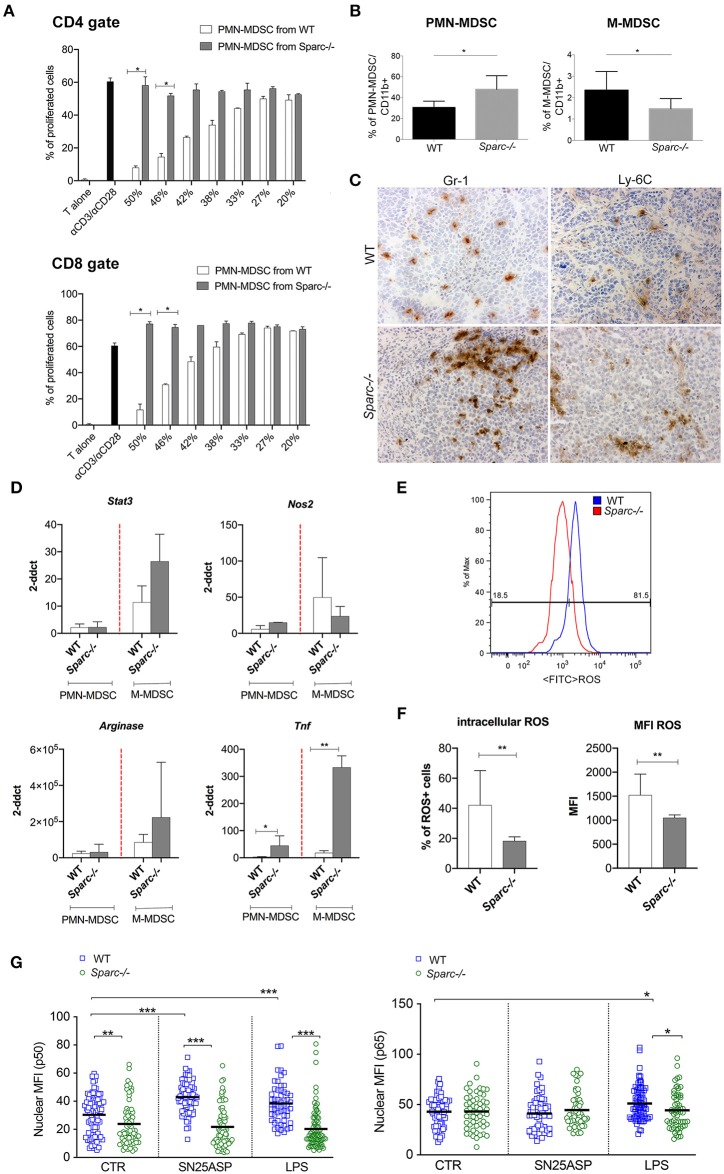
SPARC-deficient MDSC are less suppressive than WT counterparst. **(A)** Immunosuppressive activity of PMN-MDSC isolated from the spleens of WT and *Spar*c^−/−^ tumor-bearing mice evaluated as the ability to suppress a-CD3/a-CD28-induced CD4 and CD8 T cell proliferation *in vitro*. **(B)** FACS analysis of CD11b^+^, PMN- and M-MDSC performed on peripheral blood of WT and *Spar*c^−/−^ mice injected with the SN25ASP cell line. The Student's t test was used for statistical analysis (*p < 0.05). **(C)** IHC analysis of the myeloid markers Gr-1 and Ly-6C performed on WT and *Sparc*^−/-^ tumors, showing the enrichment in Gr-1+ cells in *Spar*c^−/−^ tumors. Scale bars, 100 mm. **(D)** Semiquantitative real-time PCR analysis for *Stat3, Arginase1, Nos2* and *Tnf* genes performed on PMN-MDSC and M-MDSC subsets sorted from SN25ASP tumors grown in WT and *Spar*c^−/−^ mice (*n* = 4 for per group). The Student's t test was used for statistical analysis (*p < 0.05; **p < 0.01). **(E)** Representative histogram plots for ROS detection in WT and *Spar*c^−/−^ MDSC. Oxidation of the cell-permeant dye by ROS generate a bright green fluorescence detectable at FACS in the FITC channel. **(F)** Cumulative day showing ROS production by MDSC in terms of percentage of cells oxidating the dye and therefore expressing ROS or the MFI of expression of the oxidated permanent dye. (Student *t*-test **p < 0.01). **(G)**. Quantitative data showing p50 and p65 nuclear translocation in MDSC differentiated in presence of G-CSF, GM-CSF, and IL-6 from the BM of WT and *Spar*c^−/−^ mice. MDSC were culture for 2 h in presence of SN25ASP tumor supernatants or LPS (*p < 0.05; ***p < 0.001).

To test the functional relevance of SPARC expressed by MDSC, PMN- and M-MDSC subsets were purified from the spleen of tumor-bearing WT or *Spar*c^−/−^ mice and evaluated for their capacity to inhibit T cell proliferation *in vitro*. PMN-MDSC from *Spar*c^−/−^ mice had a marked reduced ability to suppress T cell proliferation (Figure 3A). Comparison between PMN- and M-MDSC subsets, in this model, was however cumbersome being PMN-MDSC the population that expands mostly in the spleen of tumor-bearing mice, accounting for nearly 80% (77.8 ± 9.6) of the CD11b^+^ cells, in comparison to the M-MDSC that account for 1% (1.10 ± 0.3). Additionally, the expansion of PMN-MDSC (Ly6G^high^ cells) in *Spar*c^−/−^ hosts was even higher than in WT counterparts, whereas the M-MDSC (Ly6C ^high^ cells) fraction was reduced (Figures 3B,C).

To explain the reduced suppressive capacity of PMN-MDSC from *Spar*c^−/−^ vs. WT mice, we evaluated the expression of genes that are involved in MDSC suppressive activity in FACS-sorted MDSC from both tumors and spleens (29). Despite their paucity, we also included FACS-sorted M-MDSC obtaining enough material at least for RT-PCR analysis.

Expecting differences, we were surprised of finding similar or higher expression of *Stat3* and *Arginase-I* in *Spar*c^−/−^ MDSC (both from spleen and tumor) (Figure 3D and Supplementary Figure 2). Different, however, was *Nos2* that was higher in *Spar*c^−/−^ than WT PMN-MDSC. NO, the product of NOS2 activity, is a well-recognized pro-inflammatory agent involved, for example, in ulcerative colitis. In support of this idea, *Tnf* mRNA level was higher in *Spar*c^−/−^ than WT MDSC (Figure 3D).

Trying to explain the reduced suppressive activities of Sparc^−/−^ MDSC we evaluate ROS expression in total MDSC (CD11b+ fraction, as described in Melani et al. (6) isolated from the spleen of WT and *Spar*c^−/−^ tumor-bearing mice. We found a significantly decreased ROS expression by MDSC isolated fro *Spar*c^−/−^ hosts (Figures 3E,F).

To further study the mechanisms behind SPARC induction of a pro-tumoral phenotype in MDSC, we look at NF-kB signaling, as this pathway is involved in monocytes to M-MDSC reprogramming (30–32). To test whether *Spar*c^−/−^ MDSC have defective NF-kB activation, we evaluated p65 and p50 translocation into the nucleus of BM-differentiated MDSC (28) from WT and *Spar*c^−/−^ mice, after exposure to tumor supernatants or, as control, to LPS. Confocal microscopy analysis revealed that SPARC-deficient MDSC showed a significantly lower amount of p50 but not of p65 into the nucleus (Figure 3G and Supplementary Figure 3) than SPARC-competent MDSC, at baseline or when in culture with SN25ASP tumor supernatant (Figure 3G). This suggests that SPARC-deficient PMN-MDSC may be skewed toward an inflammatory phenotype.

### Endogenous SPARC Reverts Tumor-Induced PMN Education Toward Cytostasis

**Figure 4:**
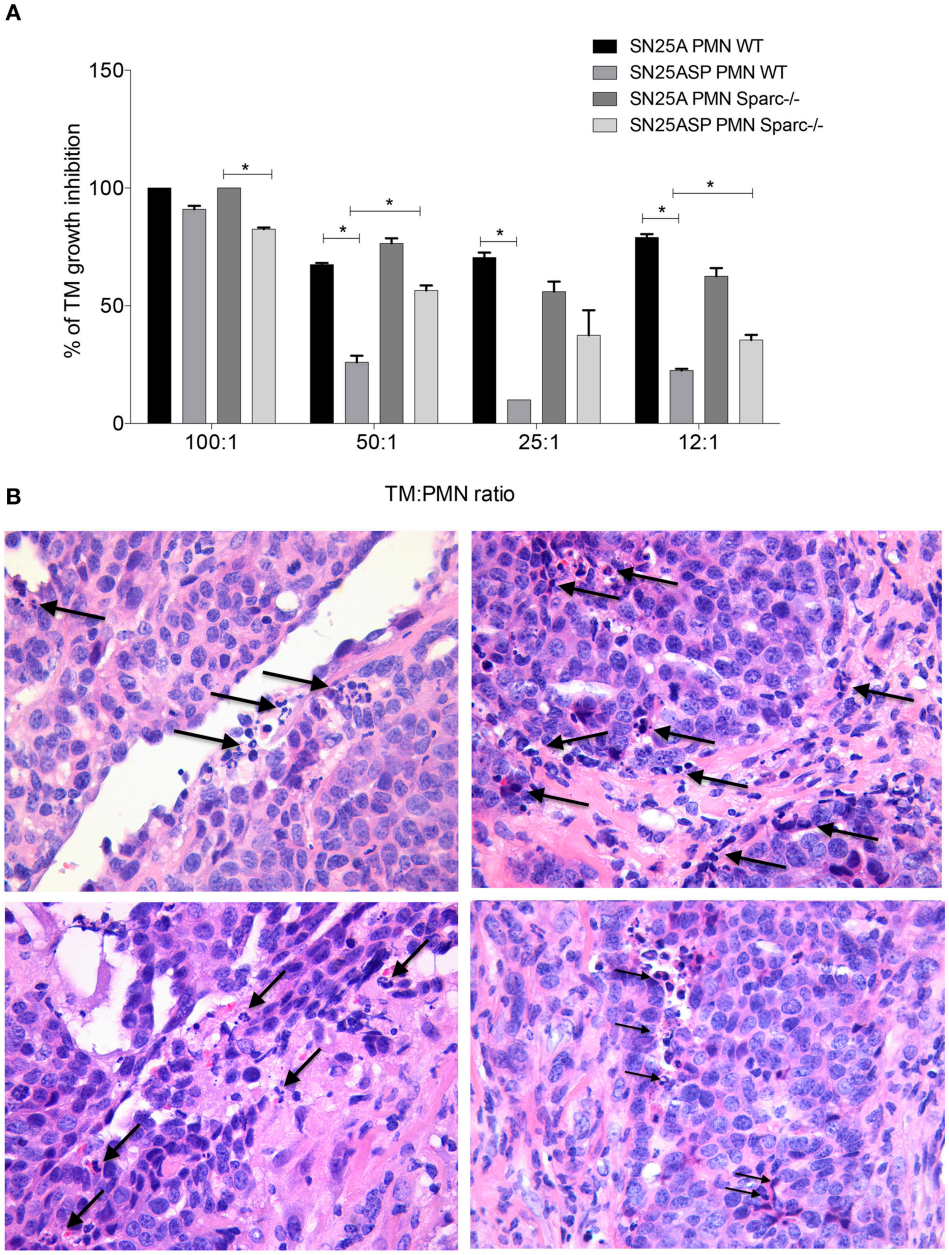
In the absence of SPARC PMN display increased cytostatic activity over tumor cells. **(A)** PMN-mediated cytostatic activity against SN25A and SN25ASP cells. Bars represent the PMN-mediated growth inhibition, means +/- SD of triplicate are shown (*p < 0.05). **(B)** H&E analysis showing PMN infiltrating granulocytes undertaking lytic activity (arrows) on both tumor cells and vascular structures.

Inflammatory PMN, isolated from subcutaneous agar-implants, exert cytostatic activity toward G-CSF releasing tumor cells (17). Using inflammatory PMN isolated from WT and *Spar*c^−/−^ mice, we evaluated whether tumor cells, according to their ability to promote MDSC differentiation (SN25ASP > SN25A), can inhibit such PMN function and whether PMN from SPARC-competent and -deficient mice are differently susceptible to tumor induced re-education and therefore capable of different cytostatic activity on tumor cells. To this end PMN from WT or *Spar*c^−/−^ mice, were co-cultured with SN25A or SN25ASP tumor cells. Both SN25A and SN25ASP cells lines express G-CSF at similar level, whereas they release different amounts of GM-CSF, IL-6 and COX-2, all significantly higher in the SPARC-over-expressing cell line (23). Co-culturing these cells with PMN we found that WT PMN had cytostatic activity only against SN25A cells, whereas they were significantly less cytostatic when co-cultured with SN25ASP cells (Figure 4A). *Spar*c^−/−^ PMN were similarly able to inhibit the growth of SN25A cells (Figure 4A) but contrarily to WT PMN, were also able of cytostasis against SN25ASP cells (Figure 4A). These results suggest that SPARC, endogenously produced by MDSC, contributes to tumor-induced education of myeloid cells toward a pro-tumoral phenotype. In line with the pro-inflammatory, anti-tumor activity of myeloid cells from *Sparc*-deficient mice, histological analysis of SN25ASP grown in *Spar*c^−/−^ mice showed features of stromal and vascular damages with vascular lacunae characterized by infiltrating granulocytes undergoing lytic activities (Figure 4B).

### Altered Tumor Vascularization in SPARC-Deficient Hosts

**Figure 5:**
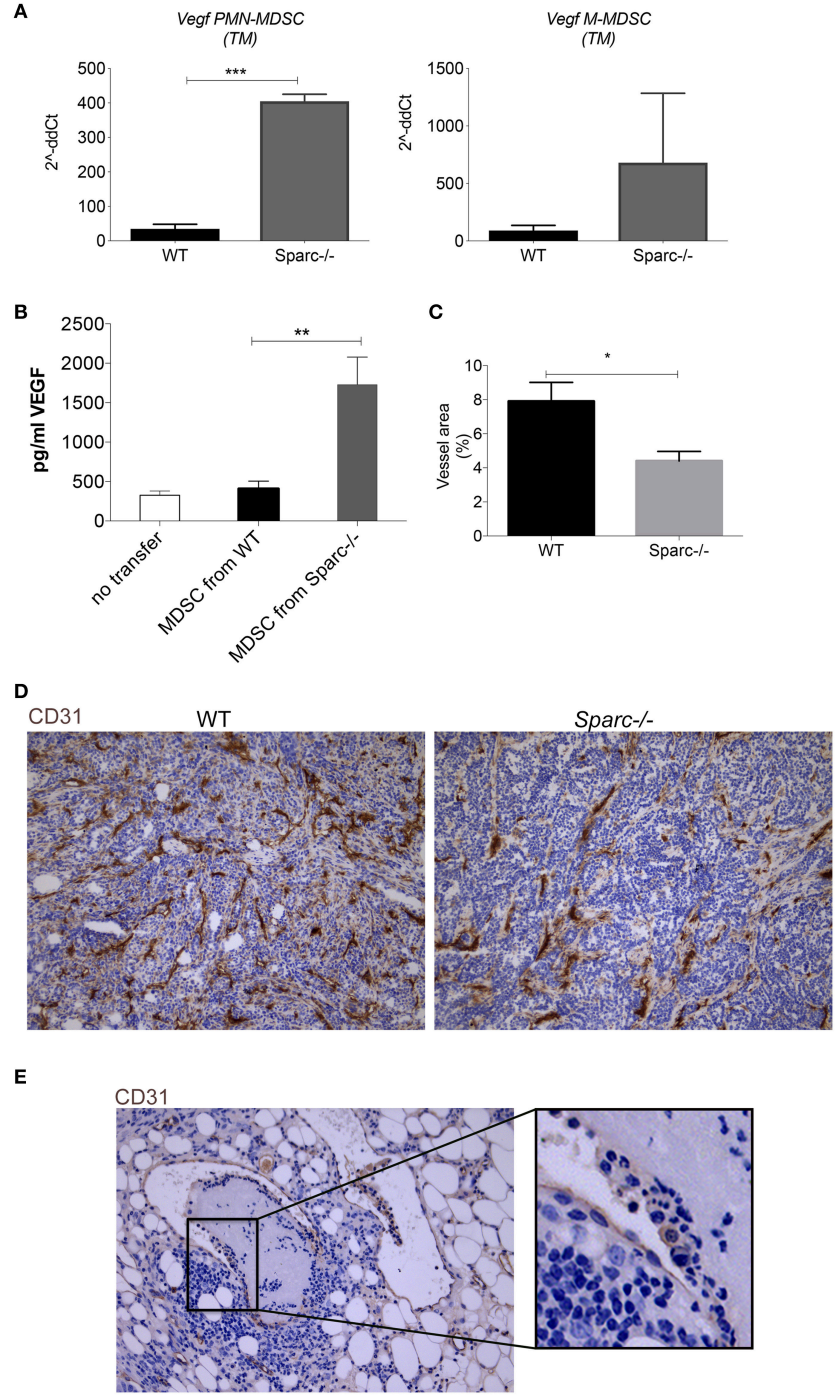
Increased VEGF expression in MDSC from Sparc^−/−^ mice. **(A)** Semiquantitative real-time PCR analysis for *Vegf* performed on PMN-MDSC and M-MDSC subsets sorted from SN25ASP tumors grown in WT and *Spar*c^−/−^ mice (*n* = 4 for per group). The Student's *t*-test was used for statistical analysis (***p < 0.001) **(B)** Representative IHC analysis for CD31 of SN25ASP tumors grown in WT and *Spar*c^−/−^ mice (*p < 0.05; **p < 0.01) **(C)** Quantification of the vessel areas calculated as (CD31+ area/total tumor area)*100. **(D)** Representative IHC analysis for CD31 in SN25ASP tumor sections from *Spar*c^−/−^ mice. **(E)** The representative picture highlights the presence of PMN (box) destroying the vessel wall.

MDSC can support tumor growth also promoting angiogenesis, for example through the production of VEGF. We investigated whether SPARC can influence tumor angiogenesis as part of their pro-tumorigenic activities. We evaluated VEGF expression by RT-PCR in spleen and tumor MDSC from WT and *Spar*c^−/−^ mice and the serum level of VEGF in SN25ASP tumor-bearing *Spar*c^−/−^ mice, receiving or not a transfer of WT or *Spar*c^−/−^ MDSC. *Vegf* mRNA expression was higher in *Sparc*-deficient PMN-MDSC and M-MDSC (Figure 5A), as it was the amount of VEGF in the serum of *Spar*c^−/−^ mice injected with *Sparc*-deficient rather than WT MDSC (Figure 5B), suggesting that the lack of SPARC in MDSC may favor tumor vascularization. However, IHC analysis of tumor sections showed a reduced staining of CD31, a marker of endothelial cells, in *Spar*c^−/−^ than in WT mice (Figures 5C,D). Therefore, despite a potential increase in tumor angiogenesis because of higher VEGF availability, the concomitant pro-inflammatory nature of *Spar*c^−/−^ PMN likely limits the formation of an efficient vascular network. In favor of this interpretation, IHC analysis performed onto tumor sections shows the presence of PMN destroying the vascular wall of CD31^+^ vessels (Figure 5E). Overall these results point to less suppressive *Spar*c^−/−^ MDSC, endowed with cytostatic activities, and of the capacity of damaging tumor vasculature, thus explaining the reduced growth of tumor implanted into *Spar*c^−/−^ mice.

### The Absence of SPARC Increases Neutrophil Extracellular Trap Extrusion by PMN-MDSC

**Figure 6:**
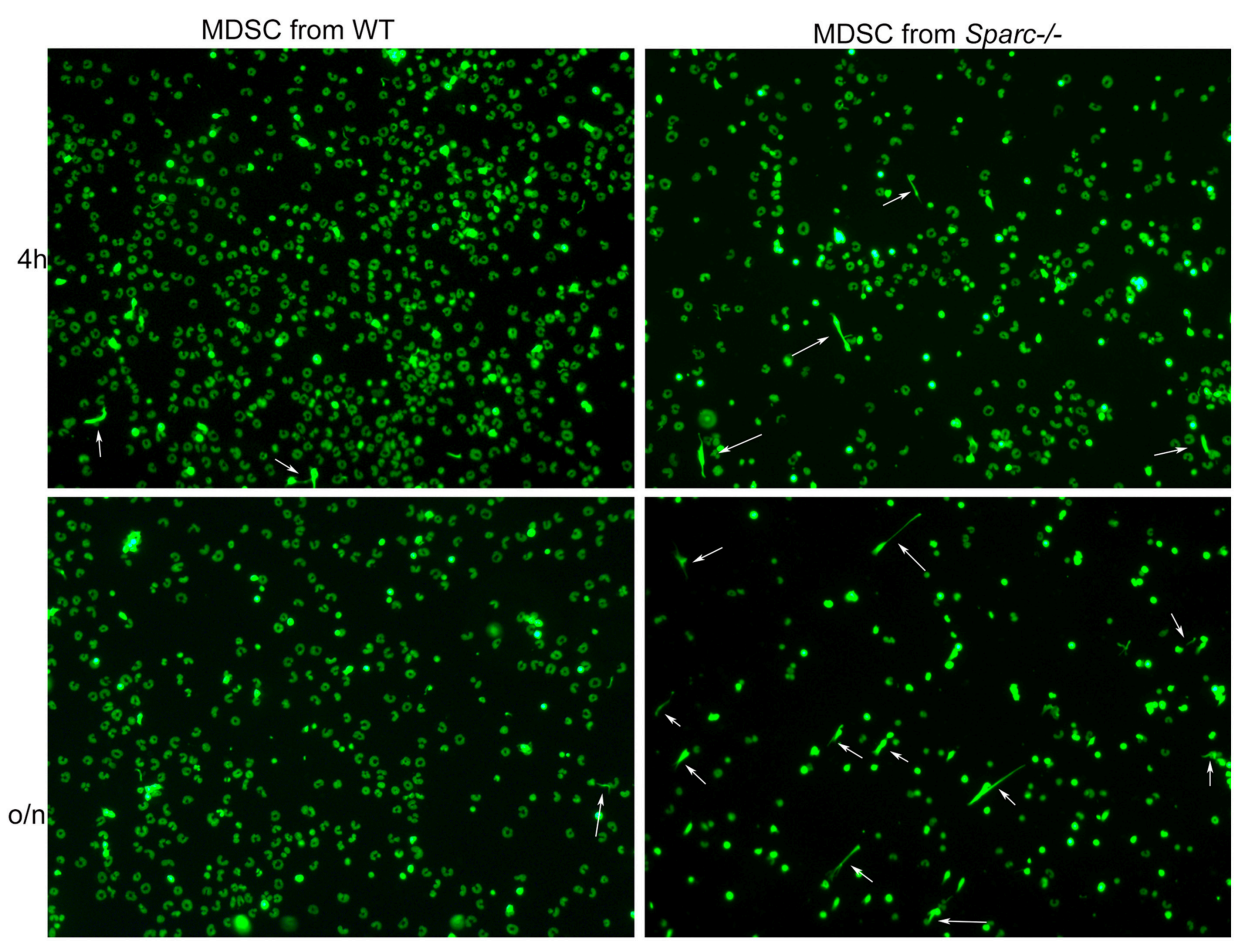
Increased NET formation by Sparc^−/−^ MDSC in comparison to WT counterpart. Representative IF analysis showing increased presence of NETs (white arrows) in *Spar*c^−/−^ MDSC seeded onto poly-D-Lysine coated glasses in presence of the DNA dye Sytox green.

NETs are extruded by activated PMN after exposure to a variety of factors (i.e., immune complexes, IFNs, TNF and others). Recently it has been shown that NETs can be extruded by MDSC within the TME via IL-8 stimulation (33). This finding prompted us to assess whether the absence of SPARC could impact NET formation by MDSC expanded in presence of a tumor. To this end PMN-MDSC were isolated from the spleen of tumor-bearing *Spar*c^−/−^ and WT mice, seeded onto poly-D-Lysine coated glasses and stimulated or not with PMA to induce NETosis. We observed that MDSC from tumor-bearing WT and *Spar*c^−/−^ mice were equally able to extrude NETs in presence of PMA, but in its absence only MDSC isolated from *Spar*c^−/−^ mice were able to extrude NETs (Figure 6). This might suggest that the sensing of different specific factors or the lack of some brakes produced *in vivo* in the absence of SPARC could differently prime PMN-MDSC for NET formation.

## Discussion

Myeloid cells expand within the bone marrow and migrate into the periphery where they are skewed toward MDSC.

We propose that SPARC expression in MDSC is required for their pro-tumor “education.” In the absence of endogenous SPARC, MDSC are indeed less suppressive and have reduced capacity to sustain EMT and tumor outgrowth. The reduced suppressive capacity was particularly evident on PMN-MDSC. However, due to the very low amount of M-MDSC in our mammary tumor models we were unable of testing whether SPARC could also influence the activity of the monocytic subset. Although not subverting numerically the PMN-MDSC, in the majority of mouse tumor models a prevalent function is given to M-MDSC (34). However, in few cases the relevance of the suppressive activity of PMN-MDSC has been clearly shown (4). Furthermore, other matricellular proteins have been demonstrated relevant for immunosuppression, such as osteoactivin, also known as glycoprotein nonmetastatic B (GPNMB) (35) and intracellular ostepontin (iOPN) (27).

Different transcription factors have been involved in the acquisition of MDSC suppressive phenotype, among which the best-characterized are STAT3, STAT1 and NF-kB. STAT3 works preferentially on PMN-MDSC and is largely involved in MDSC expansion. In our models, Arginase-I and NOS2, whose expression can be controlled by STAT3, were surprisingly not down-regulated in *Spar*c^−/−^ MDSC, which are low suppressive. Flow cytometry analysis showed equal phosphorylation of STAT3 in WT and *Spar*c^−/−^ MDSC. Also, STAT1, which contributes to suppression, was equally phosphorylated in MDSC from the two strains. Although in contrast with the expected immune suppression, the high STAT3 pathway found in *Spar*c^−/−^ MDSC is in line with the increased VEGF that is regulated by, but also regulate, STAT3 (36, 37). We have already described that VEGF expands MDSC suggesting that STAT3 may concur to both suppression and expansion of MDSC, but through uncoupled mechanisms (6). Thus, in *Spar*c^−/−^ mice MDSC expansion can largely depend on VEGF, whereas other mechanisms can account for the reduced MDSC suppression, in presence of key mediators like *Stat3, Nos2*, and *Arginase1*. Searching for possible relevant differences between *Spar*c^−/−^ and WT MDSC we found reduced nuclear translocation of the NF-kB p50 subunit, in the former. This may suggest that reduced level of p50 subunits may limit the formation of immunosuppressive p50:p50 homodimers in favor of the p65:p50 inflammatory heterodimers. Supporting this hypothesis, the production of TNF by *Spar*c^−/−^ MDSC was significantly higher than by WT MDSC. Furthermore, very recently Veglia *et al*. reported that the deletion of the fatty acid transport protein 2 (FATP2) abrogated the suppressive activity of PMN-MDSC leaving unaffected the expression of *Arginase 1* and *Nos2* (4). This discrepancy in expression of suppressive genes and MDSC suppressive activity was explained showing reduced PGE2 production by PMN-MDSC from *Fatp2-KO* compared to WT mice. We previously shown that the intracellular retention of SPARC in tumor cells through the over-expression of SCD5, an enzyme that mediated the synthesis of monounsatured fatty acids (MUFA), suppressed tumor growth through an alteration of satured and monounsatured FA balance. The last impacted on tumor growth and metastasis (38). It is reasonable that knocking-down SPARC in MDSC would result in an alteration of FA balance that might ultimately impact on PMN-MDSC functions, as occurred in the case of *Fatp2-KO* mice. Differently from Veglia et al. (4) we found that PMN-MDSC isolated from the spleen of tumor-bearing WT and *Spar*c^−/−^ mice showed a strongly reduced ROS expression in those from *Spar*c^−/−^ mice. However, our findings are in line with the role of fatty acids in the induction of cytosolic and mitochondrial reactive oxygen species (ROS) (38).

Overall these results suggest that the reduced ROS expression combined to the high production of TNF could account for the anti-tumor activity of *Spar*c^−/−^ myeloid cells.

In fact although associated with tumor-induced chronic inflammation (39), TNF if produced at high doses becomes a key factor in mediating tumor-rejection (40). Therefore, it is foreseeable that an unbalance in TNF production could skew MDSC toward an inflammatory, anti-tumor phenotype. Notably, TNF is also required for inflammation-driven NETosis. Indeed, we previously showed impaired NET formation in TNF-KO mice (16) and high TNF in *Spar*c^−/−^ MDSC might explain their increased spontaneous NET formation obtained *in vitro* by seeding MDSC onto poly-D-lysine coated glasses. *In vivo*, spontaneous NETosis was observed mainly in the case of SN25A, a *Sparc*-null tumor when injected into *Sparc*-deficient mice, and less in the case of SN25ASP tumors. The likely explanation should consider that NET formation is negatively regulated by collagens (via LAIR-1) and that collagen is more abundant in SPARC-transduced tumors. This context, associated with a robust inflammatory environment of *Spar*c^−/−^ mice, exacerbates NET formation and their pathogenicity *in vivo*. As occurring in systemic vasculitis (41), in which NETs promote endothelial damage, we found sign of vascular damages in tumors grown in *Sparc*-deficient mice.

Unexpectedly, despite the influence of MDSC-derived SPARC on EMT markers and immune suppression, the tumor volume of SN25ASP tumors injected in WT and Sparc ^−/−^ mice was similar at the end, although the differed kinetics of growth that was initially faster in WT mice. Several years ago we published that neutrophils can control tumor growth and favor the elicitation of anti-tumor immune responses (42). Our data suggest that PMN-MDSC from *Spar*c^−/−^ mice behave as N1-like neutrophils rather than MDSC, a condition that allows them to initially control tumor growth until other immune suppressive mechanisms take over (i.e., CD8 T cells exhaustion). Indeed, tumors injected in *Spar*c^−/−^ mice show higher infiltration by CD8 T cells characterized by the expression of multiple markers of exhaustion (not shown).

These results prompt the hypothesis that NET could come in different flavors, according to the context in which they are generated, to sustain either pro-tumor or anti-tumor immunity. An additional level of complexity is introduced by the ECM, as the amount of collagen influences NET formation, despite the presence of an inflammatory environment suitable for such an event.

In conclusion, this paper proposes SPARC as a new potential marker of MDSC, in both human and mouse, with the additional feature of controlling MDSC suppressive activity with the aim of preventing an excessive anti-tumor inflammatory state.

## Data Availability

The raw data supporting the conclusions of this manuscript will be made available by the authors, without undue reservation, to any qualified researcher.

## Ethics Statement

The study on human samples was approved by the Medical Ethics Committee (Auth. Number 167/17), and all clinical data were obtained after receiving informed consent, according to institutional rules. All experiments involving animals were approved by the Ministry of Health (INT 16_2016, authorization number 288/2017-PR).

## Author Contributions

SS and MC designed the research. GT, CC, BC, LB, PP, AG, and FC performed the experiments. MD and GR provided human blood samples and analyzed clinical parameters. SS, GT, AS, CT, CC, and SS analyzed the data. SS, CC, and MC wrote the manuscript.

### Conflict of Interest Statement

The authors declare that the research was conducted in the absence of any commercial or financial relationships that could be construed as a potential conflict of interest.
