# Supplementary material for: SPARC Is a New Myeloid-Derived Suppressor Cell Marker Licensing Suppressive Activities
Source: Front Immunol. 2019 Jun 20;10:1369. doi: 10.3389/fimmu.2019.01369 (PMC6596449; doi:10.3389/fimmu.2019.01369)
Supplement: Supplementary file 1 [file Data_Sheet_1.docx]

**Supplementary Figures**

*Supplementary Figure 1*


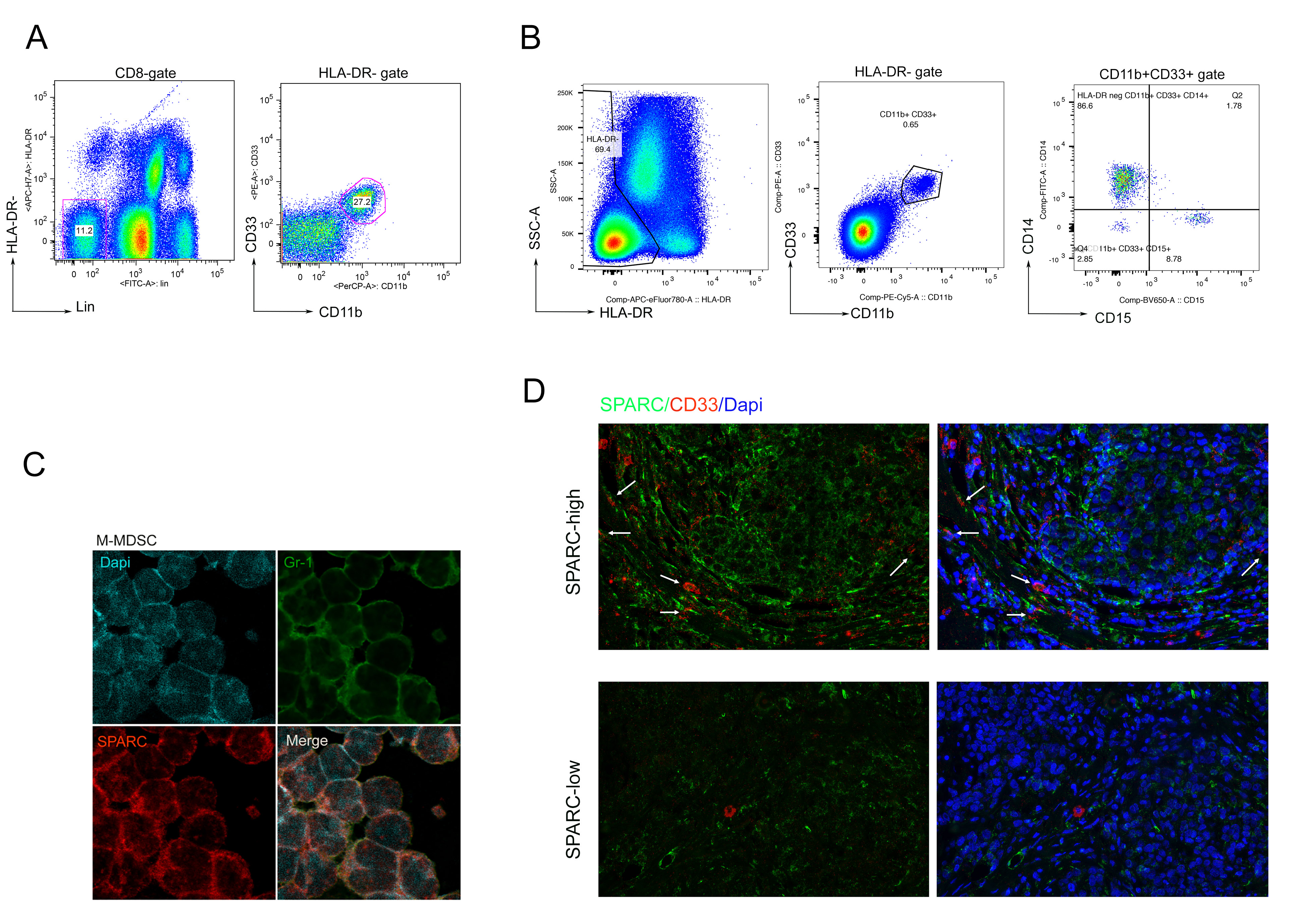


**A.** Gating strategy for the identification of eMDSC subset in the peripheral blood of breast cancer patients. CD11b+CD33+ eMDSC were defined within the gate of HLA-DR-Lin- cells. HLA-DR-Lin- cells were identified within the CD8- cells gate. CD8+ gate was determined on live cells after the exclusion of doublets. **B.** Gating strategy for the identification of PMN- and M- MDSC subset in the peripheral blood of breast cancer patients. PBMC were stained with mAB to: HLA-DR, CD33, CD11b, CD14 and CD15. HLA-DR-CD33. HLA-DR-CD33+CD11b+CD15+ PMN-MDSC and HLA-DR-CD33+CD11b+ CD14+ M-MDSC were identified within the CD11b+CD33+ gate. The CD11b+CD33+ gate was defined on HLA-DR- cells. HLA-DR+ cells were identified within the gate of live cells after doublets exclusion.

**C**. Cytospin preparations of FACS-sorted M-MDSC isolated from SN25ASP tumors and stained for Gr1 (green) and SPARC (red).

**D.** Representative confocal microscopy analysis showing the increased recruitment of CD33+ cells in human BC tumors in which SPARC was highly expressed by the tumor cells. On the contrary TM characterized for low SPARC expression were associated to a reduced recruitment of CD33+ cells that never express SPARC. On the contrary in SPARC-high tumors associated CD33+ cells, at least in part, express SPARC (white arrows).

*Supplementary Figure 2*


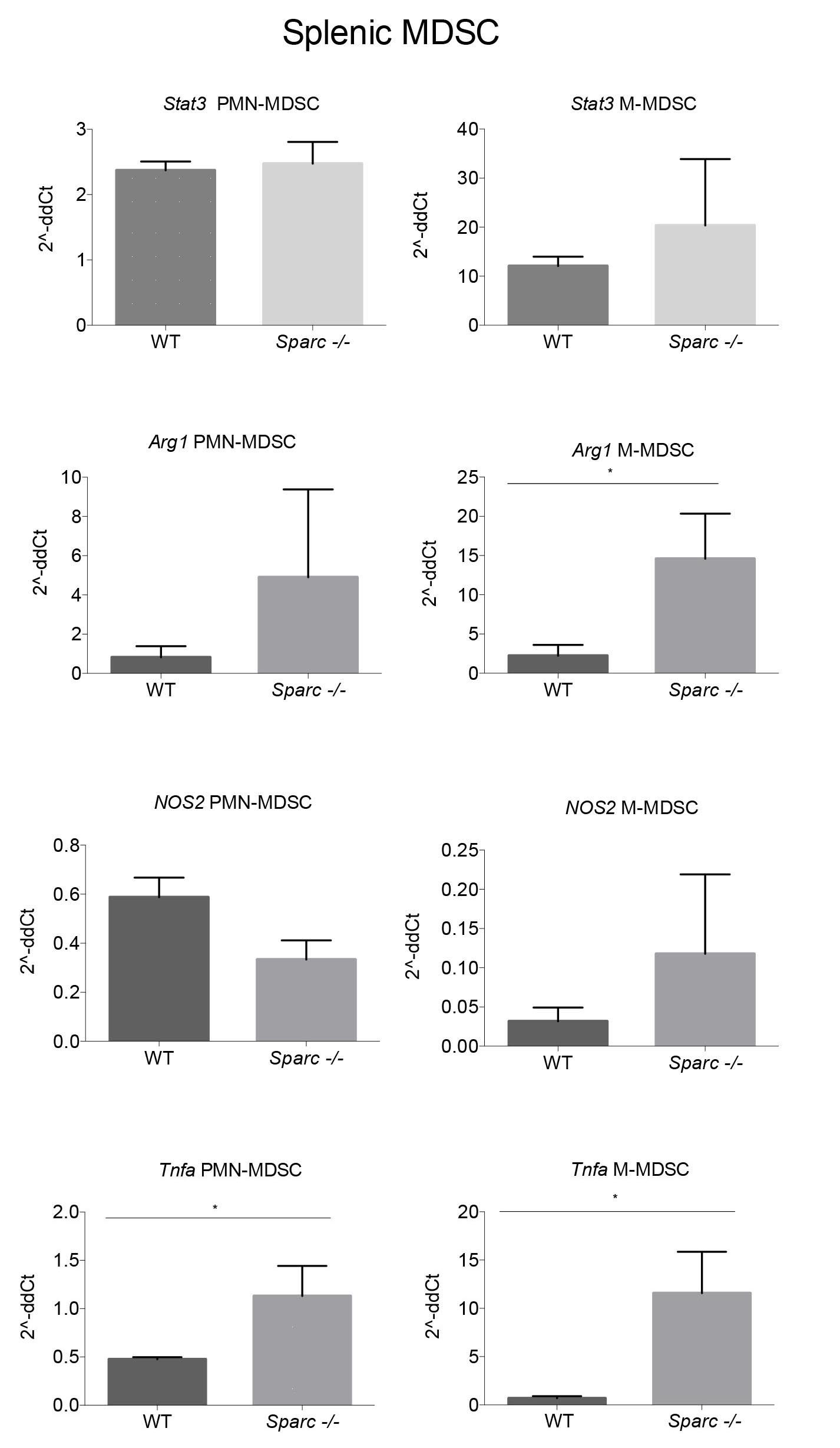


Semiquantitative real-time PCR analysis for *Stat3, Arginase1, Nos2* and *Tnf* genes performed on PMN-MDSC and M-MDSC subsets sorted from the spleen of WT and *Sparc-/-* mice bearing SN25ASP tumors (n = 4 for per group). The Student’s t test was used for statistical analysis (*p < 0.05).

*Supplementary Figure 3*


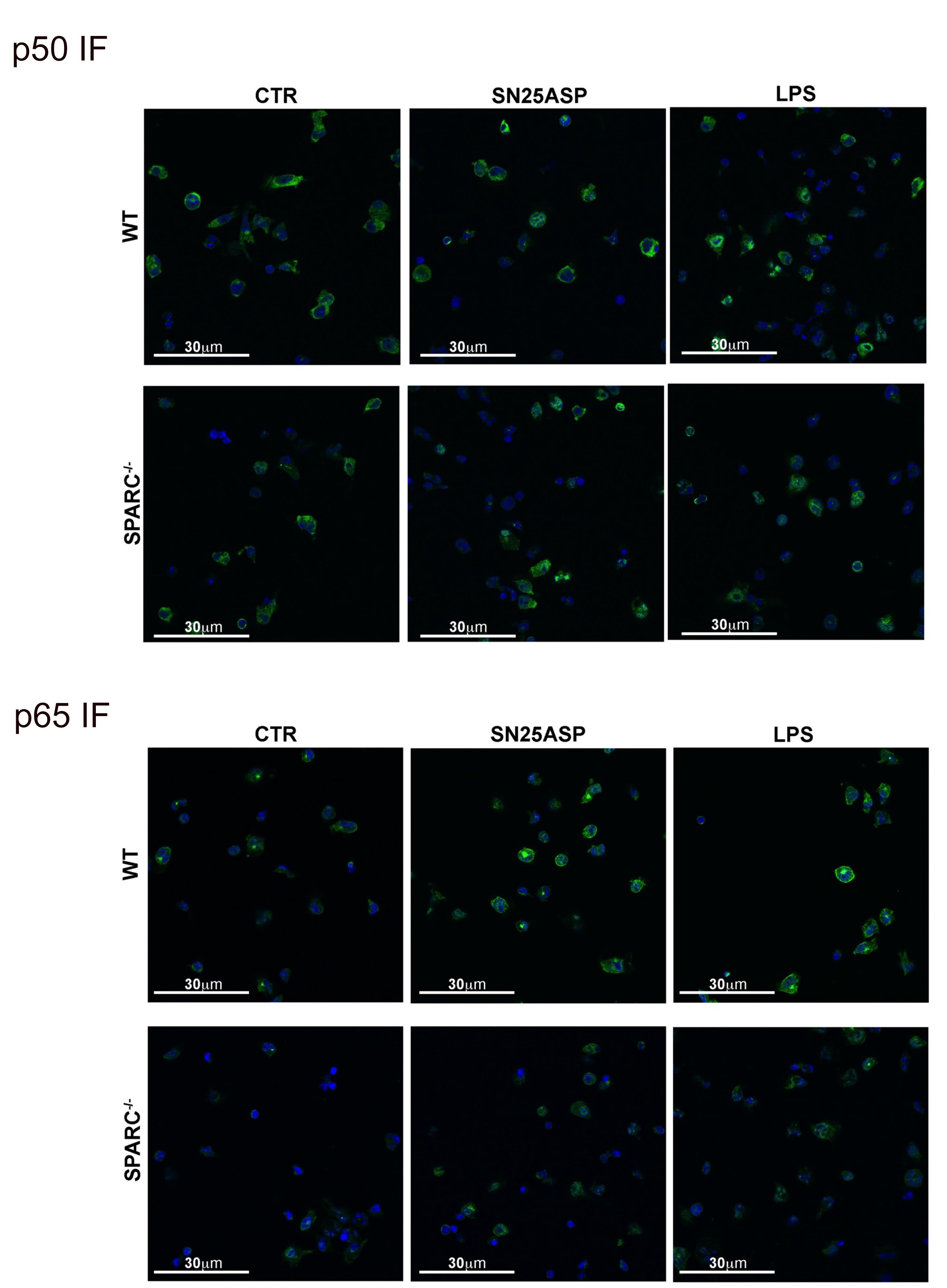


Representative confocal microscopy analysis for p50 and p65 performed on BM-derived MDSC obtained from WT and *Sparc-/-* mice and cultured 2h in presence of SN25ASP tumor supernatants or LPS (10 ng/ml).

**Supplementary Table 1.** Antibody used for flow cytometry

| Fluorescence | Antigen | Clone | Manufacturer |
| --- | --- | --- | --- |
| Mouse TM ANALYSIS | | | |
| FITC | **CD45** | 30-F11 | BD |
| Pe-Cy7 | **CD11b** | M1/70 | Tonbo Biosciences |
| APC | **Ly6C** | AL-21 | BD |
| PE | **Ly6G** | 1A8 | Tonbo Biosciences |
| Mouse SPLENIC ANALYSIS | | | |
| FITC | **CD11b** | M1/70 | eBioscience |
| APC | **Ly6C** | AL-21 | BD |
| PE | **Ly6G** | 1A8 | Tonbo Biosciences |
| Human ANALYSIS | | | |
| FITC | **CD14** | 61D3 | eBioscience |
| BV650 | CD15 | HI98 | BD |
| PE | **CD33** | HIM3-4 | BD |
| BB700 | **CD11b** | D12 | BD |
| APC-Cy7 | **HLA-DR** | G46-6 | BD |
| FITC | **Lin1** |  | BC |

| Antigen | Type | Manufacturer |
| --- | --- | --- |
| ZEB1 (NBP1-05987) | Rabbit | Novus |
| SPARC | Goat | R&D |
| E-Cadherin (ECCD-2) | Rat | Calbiochem |
| Ly6C (ER-MP20) | Rat | Acris |
| Gr-1 (RB6-8C5) | Rat | BD |
| CD31 (ab28364) | Rabbit | Abcam |
| NF-kB p50 (Sc-114) | Rabbit | Santa Cruz |
| NF-kB p65 (pSer536) | Monoclonal anti-mouse | Novus |

**Supplementary Table 2**. Antibodies used for IF and IHC
